# Engineering of chimeric natural killer cell receptors to develop precision adoptive immunotherapies for cancer

**DOI:** 10.1111/cei.13478

**Published:** 2020-07-25

**Authors:** J. Obajdin, D. M. Davies, J. Maher

**Affiliations:** ^1^ School of Cancer and Pharmaceutical Sciences CAR Mechanics Laboratory Guy’s Cancer Centre King’s College London London UK; ^2^ Department of Clinical Immunology and Allergy King’s College Hospital NHS Foundation Trust London UK; ^3^ Department of Immunology Eastbourne Hospital Eastbourne UK; ^4^ Leucid Bio Ltd Guy’s Hospital London UK

**Keywords:** cancer, immunotherapy, CAR, natural killer receptors

## Abstract

Natural killer (NK) cells are innate immune effectors which play a crucial role in recognizing and eliminating virally infected and cancerous cells. They effectively distinguish between healthy and distressed self through the integration of signals delivered by germline‐encoded activating and inhibitory cell surface receptors. The frequent up‐regulation of stress markers on genetically unstable cancer cells has prompted the development of novel immunotherapies that exploit such innate receptors. One prominent example entails the development of chimeric antigen receptors (CAR) that detect cell surface ligands bound by NK receptors, coupling this engagement to the delivery of tailored immune activating signals. Here, we review strategies to engineer CARs in which specificity is conferred by natural killer group 2D (NKG2D) or other NK receptor types. Multiple preclinical studies have demonstrated the remarkable ability of chimeric NK receptor‐targeted T cells and NK cells to effectively and specifically eliminate cancer cells and to reject established tumour burdens. Importantly, such systems act not only acutely but, in some cases, they also incite immunological memory. Moreover, CARs targeted with the NKG2D ligand binding domain have also been shown to disrupt the tumour microenvironment, through the targeting of suppressive T regulatory cells, myeloid‐derived suppressor cells and tumour vasculature. Collectively, these findings have led to the initiation of early‐phase clinical trials evaluating both autologous and allogeneic NKG2D‐targeted CAR T cells in the haematological and solid tumour settings.

## Graphical abstract

Natural killer (NK) cells are innate immune effectors which play a crucial role in recognising and eliminating virally infected and cancerous cells. This article reviews strategies used to engineer chimeric antigen receptors whereby specificity is conferred by activating NK cell receptors targeting ligands commonly upregulated on cancer cells. These CARs are expressed in T cells or NK cells for use in adoptive immunotherapy.

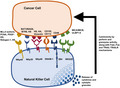

## Introduction

Transformed cells commonly acquire the ability to evade immunological detection, considered the eighth hallmark of cancer [1]. Appreciation of the fact that malignant cells are susceptible to immune surveillance has prompted the development of therapies that amplify these processes. Adoptive immunotherapy attracts particular interest, as CD8^+^ T cells and natural killer (NK) cells play a key role in inducing cancer cell death [2]. Initial studies focused on *ex‐vivo* expansion and transplantation of autologous cytotoxic T lymphocytes (CTL) and tumour‐infiltrating lymphocytes (TILs) [3, 4]. These cells express T cell receptors (TCRs), which recognize peptide‐bound major histocompatibility complex (MHC) class I antigens on target cells [5]. However, this strategy is limited by the ability of tumours to down‐regulate MHC expression or processing of tumour‐associated antigens (TAAs), thereby evading CTL detection [4, 6, 7]. Conceptually, these challenges may be overcome using chimeric antigen receptor (CAR) engineered T cells.

**Fig. 1:**
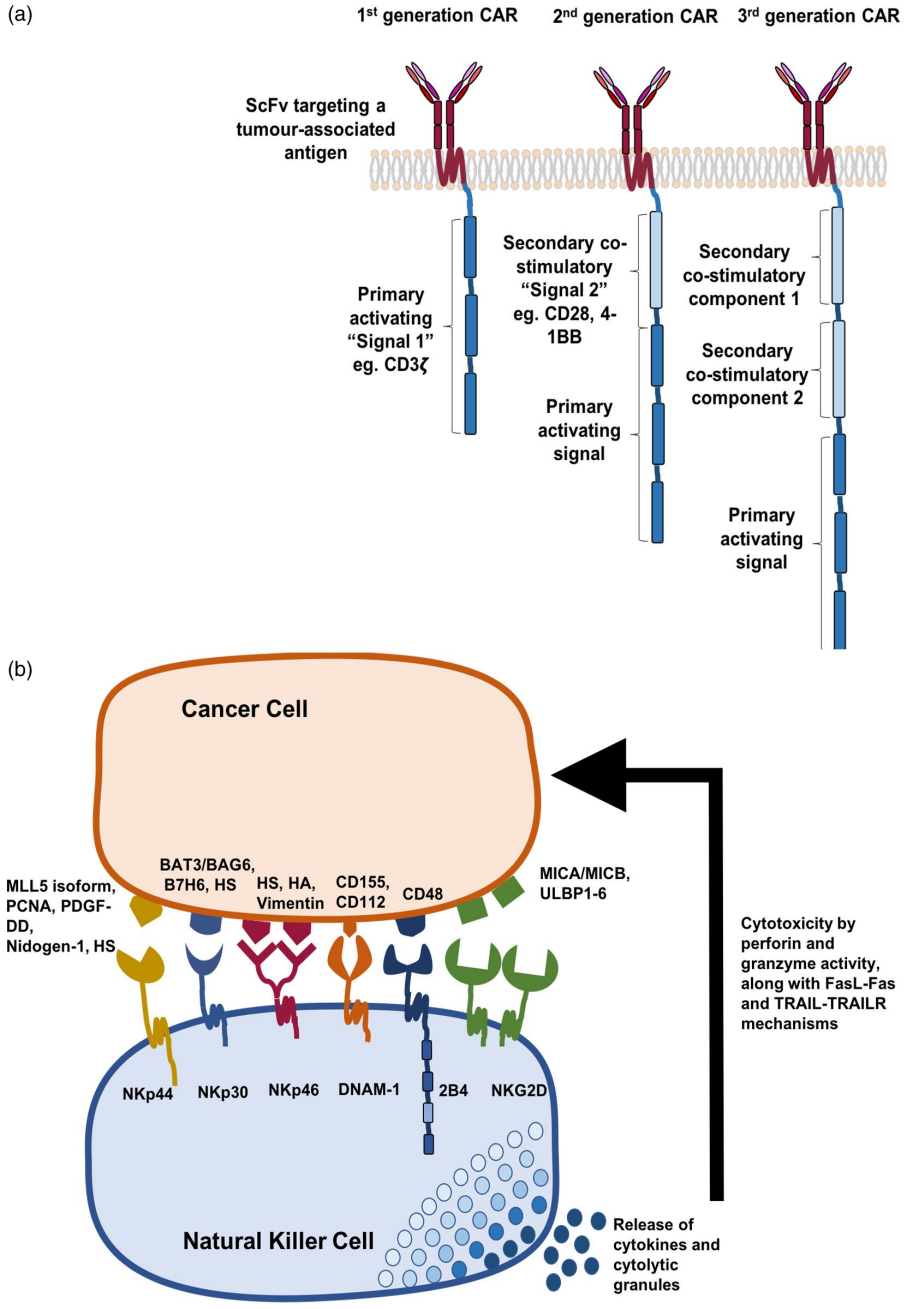
The evolution of chimeric antigen receptor (CAR) designs and the arsenal of Natural Killer cell receptors. CAR designs initially included a tumour‐antigen specific single‐chain variable fragment (scFv) fused to a primary activating signal component, typically CD3ζ, termed a first‐generation CAR. These were modified to further include one or two secondary co‐stimulatory receptor segments, such as 4‐1BB and CD28, yielding second‐ and third‐generation CARs, respectively (a). Natural killer (NK) cells are armoured with an array of receptors that relay activating signals, promoting the secretion of cytokines and cytolytic granules, mediating the lysis of tumour or virally infected cells. These receptors usually target ‘stress ligands’ commonly up‐regulated on cancerous cells, providing a favourable targeting moiety for use in redirecting T cell/NK cell specificity by means of a CAR (b). HS = heparan sulphate; HA = viral haemagglutinins.

Chimeric antigen receptors are bespoke fusion molecules that engage native cell surface‐associated TAAs without MHC restriction, coupling this to provision of T cell activating and/ or co‐stimulatory signals [5, 8, 9, 10]. Typically, CARs consist of an extracellular (EC) targeting moiety coupled via a spacer and transmembrane (TM) segment to an intracellular (IC) signalling region. The targeting moiety is typically composed of a single‐chain variable fragment (scFv) targeting a TAA, although alternative strategies include the use of short peptides, polypeptides, natural receptors and ligands, as well as modified ligands [11, 12, 13]. The precise structure of the signalling domain has given rise to a nomenclature based on CAR generations (Fig. 1a). The greatest clinical impact has been seen with second‐generation CARs, in which a single co‐stimulatory signalling component, such as CD28 or 4‐1BB, is placed upstream of an activating domain [14, 15, 16]. CAR T cell immunotherapy has achieved remarkable efficacy in relapsed/refractory B cell malignancy, indicated by the approval in multiple territories of two CD19‐targeted therapies (Kymriah and Yescarta [5]). Unfortunately, however, this approach has not proved successful for solid tumours, where poor CAR T cell migration and the suppressive tumour microenvironment (TME) hinder efficacy. Moreover, the use of highly specific scFvs imposes a further challenge, as heterogeneous target expression by tumour cells may favour antigen escape [4, 17, 18].

## NK killer cells and their receptor repertoire

The first challenge in the development of CAR T cell immunotherapy for solid tumours entails target selection. Conceptually, endogenous immune cell receptors that recognize markers of cell stress such as activating NK cell receptors may be exploited for this purpose. Balancing information transduced using activating and inhibitory innate receptors, NK cells discriminate between healthy and aberrant self [19, 20, 21]. Inhibitory receptors, including the killer immunoglobulin receptors (KIRs) and natural killer group 2A (NKG2A) in humans, recognize self‐MHC class I molecules and signal through an immunoreceptor tyrosine‐based inhibitory motif (ITIM). Although transformed and virally infected cells commonly down‐modulate self‐MHC class I expression to evade detection by T cells, NK cells recognize this loss, removing the suppressive influence of inhibitory receptors and initiating cytotoxic activity [6, 7]. In contrast, NK activating receptors recognize ligands that are specifically up‐regulated in virally infected or tumour cells, operating in an antigen‐ and MHC‐unrestricted manner. Activated NK cells can lyse target cells directly by releasing preformed cytolytic granules containing perforin and granzymes, as well as through Fas ligand (FasL) and tumour necrosis factor (TNF)‐related apoptosis inducing ligand (TRAIL). Indirect mechanisms by which NK cells induce anti‐tumour activity include the release of proinflammatory cytokines, including interferon (IFN)‐γ, tumour necrosis factor (TNF)‐α and granulocyte macrophage colony‐stimulating factor (GM‐CSF), favouring the recruitment of CTL and phagocytic cells (Fig. 1b) [22, 23].

In humans, the major activating NK receptors with prominent roles in tumour surveillance include natural killer group 2D (NKG2D), DNAX accessory molecule‐1 (DNAM‐1), the SLAM‐family receptor 2B4, the natural cytotoxicity receptors (NCRs) NKp30, NKp44 and NKp46 (Fig. 1b), as well as activating killer immunoglobulin receptors (KIRs), NKG2C‐CD94 and CD16 [19, 20, 24, 25]. Collectively, these receptors recognize multiple stress ligands specifically up‐regulated in pathogenic conditions such as infection or transformation, while being virtually undetectable in healthy tissue (Fig. 1b) [26]. Engagement of a single activating receptor is usually insufficient to trigger full NK cell activation, so these receptors interact co‐operatively [27, 28]. Complexity is heightened by the ability of many NK receptors to interact with multiple ligands, either pathogen‐derived or host genome‐encoded [29]. The crucial role of NK receptors in the recognition of several innate ‘tumour‐associated flags’ renders these molecules attractive for repurposing to target CAR T cell specificity. This concept proves favourable over the design of antigen‐specific scFvs in that affinity of the receptor–ligand interaction has been naturally optimized. In contrast, the affinity of CAR scFvs for their ligands must be carefully considered to ensure efficacy while limiting toxicity.

## Harnessing the NKG2D receptor for CAR T cell immunotherapy

The NKG2D–NKG2D ligand (NKG2DL) axis has been extensively studied in the context of immune recognition of cancer by NK cells and T cells. NKG2D is a homodimeric, C‐type lectin‐like activating receptor encoded by the *KLRK1* gene. In man, it is expressed on NK cells, CD8^+^ T cells, γδ T cells, and sometimes, CD4^+^ T cells [17, 30]. Human NKG2DL comprise two families; namely, the membrane‐spanning MHC class I‐related chains (MICA and MICB) and the glycosylphosphatidylinositol (GPI)‐linked UL16 binding proteins (ULBP1‐6; also known as retinoic acid early transcripts or RAETs) (Fig. 1b). All NKG2DL are structurally related to MHC class I molecules [17, 30, 31, 32, 33]. The murine NKG2DL include the retinoic acid early transcript (RAET) 1 proteins (RAE1α–ε), murine UL16‐binding protein‐like transcript 1 (MULT1) and the minor histocompatibility proteins, H60a‐c [32, 34]. It is noteworthy that only the ULBP/RAET genes are human–mouse orthologues. There are no mouse equivalents of human MICA or MICB, while neither H60 nor MULT1 are found in man. NKG2DL are highly polymorphic proteins, particularly MICA and MICB [34]. Affinity of interaction between NKG2D and its ligands ranges between 600 and 1100 nM in man and 2 to 700 nM (K_D_) in the mouse [35].

Physiologically, NKG2DL are not generally present in healthy tissues [36]. Levels are up‐regulated by ataxia telangiectasia‐mutated and ATM‐ and RAD3‐related (ATM/ATR)‐dependent signalling in response to DNA damage [11, 17, 30, 32, 36]. Most human haematological and solid tumours aberrantly express NKG2DLs [11, 36], while chemotherapy and radiation have been shown to further up‐regulate expression on tumour cells [30, 37, 38]. Importantly, highly immunosuppressive regulatory cells found within the tumour microenvironment (TME), including T regulatory cells (T_regs_) and myeloid‐derived suppressor cells (MDSCs), also express NKG2DL [38, 39].

The short intracellular domain of NKG2D renders the signalling activity of this homodimer wholly dependent upon its ability to associate with two homodimers of an adaptor protein, designated DNAX‐activating protein of 10 kDa (Dap10) [40]. The specificity of this interaction is dictated by reciprocally charged amino acids within the transmembrane regions, meaning that human NKG2D can only associate with Dap10 [33, 38, 41, 42]. Furthermore, stable cell surface expression and downstream signalling by NKG2D is dependent upon this association [17, 37, 41]. Similarly to the CD28 and ICOS co‐stimulatory receptors, Dap10 contains a YXXM motif that activates phosphatidylinositol 3′‐kinase (PI3K) signalling [38]. In human T cells, NKG2D‐Dap10 signalling does not direct cell killing but instead provides co‐stimulation [11, 33, 40, 43]. Engineering of chimeric receptors in which NKG2D is fused to an immunoreceptor tyrosine‐based inhibitory motif (ITAM)‐containing endodomain aims to directly provide such an activating signal, favouring therapeutic application [11].

**Fig. 2:**
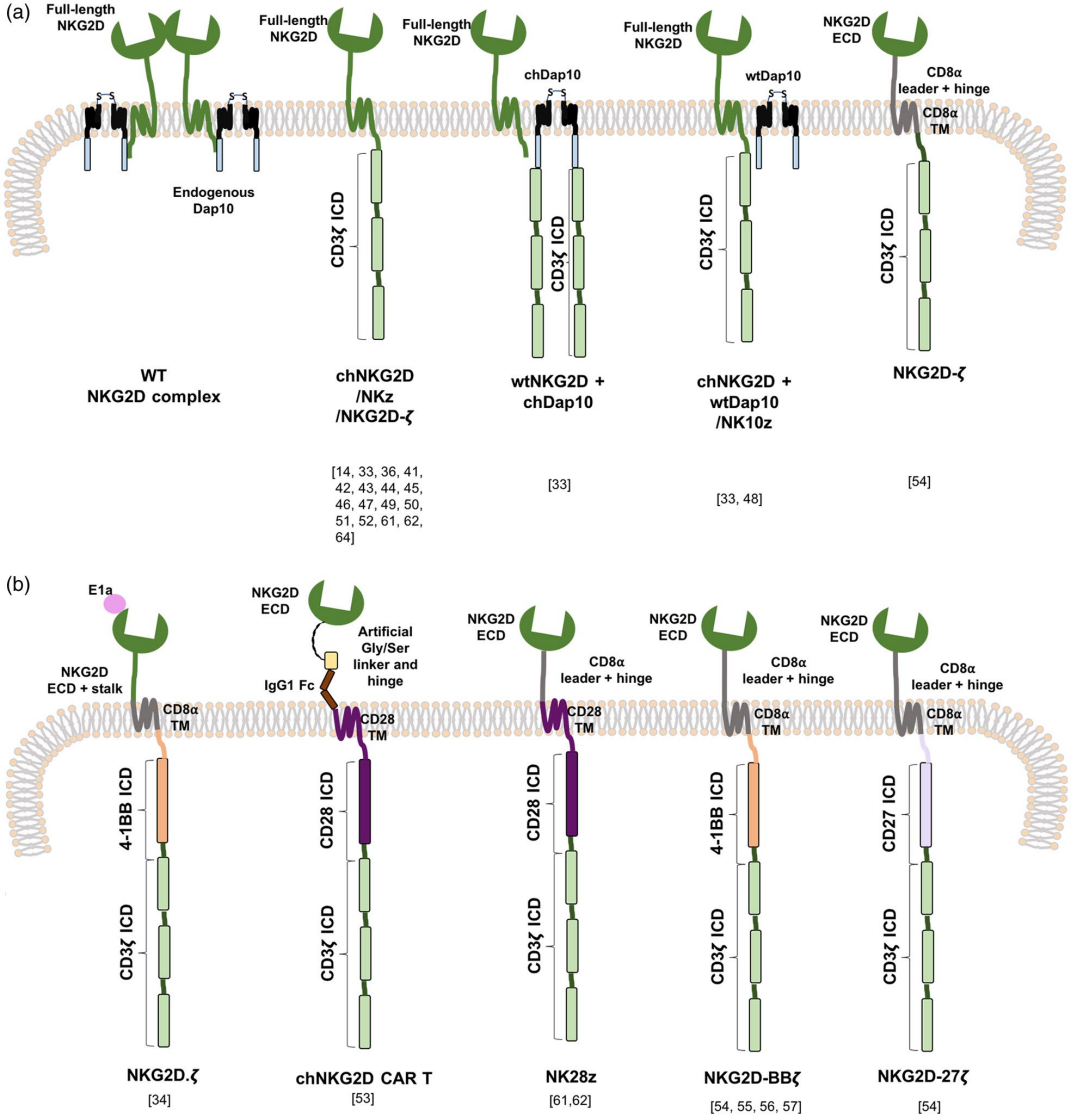
Exploiting the natural killer group 2D–natural killer group 2D ligand (NKG2D–NKG2DL) axis in chimeric antigen receptor (CAR) design. Endogenous NKG2D forms a homodimeric structure that laterally associates with two homodimers of DNAX‐activating protein of 10kDa (Dap10), forming a heterohexameric complex which initiates P13K signalling upon NKG2DL recognition. This structure was modified to include the CD3ζ endodomain, in several configurations, to mimic canonical T cell signalling upon receptor engagement while obtaining co‐stimulation from either endogenous or additional Dap10 provided (a). The NKG2D ectodomain was further evaluated in the context of second‐ and third‐generation CARs incorporating other co‐stimulatory intracellular domains (ICDs) (b). CAR constructs are shown here as monomers. However, as NKG2D forms homodimers, constructs incorporating the NKG2D ectodomain (ECD) are also expected to homodimerize. Constructs whereby CD3ζ is directly fused to NKG2D incorporate CD3ζ in reverse orientation. WT = wild‐type; TM = transmembrane.

The first engineered chimeric NKG2D receptor (chNKG2D) entailed a direct fusion of full‐length NKG2D to CD3ζ (Fig. 2a) [11]. Because NKG2D is a type 2 protein, the CD3ζ endodomain is expressed in an inverted configuration. Consequently, chNKG2D engagement initiates two signals: an activating signal through CD3ζ (‘signal 1’) and co‐stimulation delivered by endogenous Dap10‐PI3K (‘signal 2’) [11]. Enhanced cell surface CAR expression was observed when chNKG2D was co‐delivered with additional wild‐type (wt)Dap10, or when wtNKG2D was co‐expressed with both wtDap10 and a chimeric Dap10‐CD3ζ endodomain fusion (Fig. 2a) [11]. T cells expressing the wtNKG2D‐chDap10/CD3ζ and chNKG2D/CD3ζ‐wtDap10 receptor complexes efficiently lysed Rae‐1β‐bearing murine T cell lymphoma cells and secreted high levels of proinflammatory cytokines and chemokines, while sparing cells that lacked this ligand [11]. Anti‐tumour activity of chNKG2D/CD3ζ‐wtDap10 T cells was further demonstrated by the significant reduction in tumour burden in both a Rae‐1β^+^ subcutaneous [11] and systemic lymphoma model [44], without prior lymphodepleting chemotherapy. Impressively, mice remained disease‐free following subsequent tumour rechallenge, suggestive of T cell memory [11, 44]. Tumour regression was also dependent upon perforin and FasL, but not TRAIL [44]. These results were confirmed with a human chNKG2D CAR expressed in primary human T lymphocytes [45]. Soluble MICA is commonly secreted by cancer cells as an immune evasion mechanism. Nonetheless, this study demonstrated that soluble MICA only inhibited chNKG2D T cell cytotoxicity at a concentration of 15 μg/ml, significantly higher than levels detected in cancer patient sera (0·2–10 ng/ml) [45]. Similarly, an NKG2D‐4‐1BB‐CD3ζ CAR (Fig. 2b) was not inhibited when cultured in the presence of soluble NKG2DL [37].

These initial reports prompted further studies that evaluated the efficacy of chNKG2D CAR T cells against various haematological and solid tumours (Fig. 2a,b). Both allogeneic and autologous chNKG2D T cells destroyed ovarian cancer cell lines and primary human ovarian tumour cells and significantly reduced tumour burden in an *in‐vivo* syngeneic murine model [46]. Using the same model, it was found that both splenic and tumour‐infiltrating lymphocytes secreted inflammatory cytokines and chemokines. Isolated spleen cells from mice treated with chNKG2D were shown to secrete more IFN‐γ than those treated with wtNKG2D, a finding that was maintained for up to 10 weeks [39]. Splenocyte sources of IFN‐γ secretion included host NK cells, CD8^+^ and CD4^+^ T cells, indicating that these CAR T cells initiated a systemic host immune response, which itself required the presence of chNKG2D‐secreted IFN‐γ, GM‐CSF and perforin [39, 47]. This treatment further enhanced antigen processing, presentation and T cell trafficking to the tumour [47]. Moreover, it promoted the intratumoral infiltration and activation of NK cells, CD8^+^ T cells and neutrophils, while decreasing infiltrating CD19^+^ B cells and immunosuppressive forkhead box protein 3 (FoxP3^+^) T_regs_. As T_regs_, but not CD19^+^ B cells, were shown to express NKG2DL in this model, it was postulated that they were directly targeted by chNKG2D T cells [39]. Furthermore, these CAR T cells promoted the recruitment and activation of myeloid cells within the TME, enhancing their production of IFN‐γ and nitric oxide (NO). In contrast, production of T cell inhibitory factors, including interleukin (IL)‐6, IL‐10 and reactive oxygen species (ROS), were reduced [48]. These modified immunostimulatory macrophages expressed lower levels of T_reg_‐recruiting ligands and angiogenic factors, including hypoxia‐inducible factor (HIF)‐1α and vascular endothelial growth factor (VEGF) [39, 48]. chNKG2D T cells were also shown to target NKG2DL‐expressing tumour vasculature, inhibiting angiogenesis and further reducing tumour burden [49]. Collectively, chNKG2D CAR T cells not only eliminate tumour cells, but also remove immunosuppressive immune cells and promote a proinflammatory anti‐tumour systemic response dependent upon GM‐CSF and IFN‐γ [39]. Building on this, NK cells expressing a similar chNKG2D construct (Fig. 2a) caused the elimination of immunosuppressive human *ex‐vivo*‐expanded MDSCs, but not other immune cell subsets, in a xenograft tumour model [50]. Chang *et al*. also demonstrated anti‐tumour activity of NK cells that had been engineered to express chNKG2D, co‐expressed with Dap10 [51]. Cytotoxicity, induction of IFN‐γ and immunomodulatory effects of chNKG2D CAR T cells were all shown to depend upon FasL and perforin [39, 47].

The chNKG2D CAR T cell approach was also shown to be active against multiple myeloma [52]. In a syngeneic myeloma model, chNKG2D treatment significantly extended survival, enhanced by multiple sequential dosing [53]. T cells were shown to traffic to the spleen and bone marrow, and isolated splenocytes secreted IFN‐γ when co‐cultured with tumour cells used to establish disease, but not other NKG2DL^+^ tumour cells [53]. This study also demonstrated that despite poor *in‐vivo* CAR T cell persistence, a long‐term protective immune memory response, had been generated, indicated by rejection of tumour rechallenge [53]. Immunotherapy with chNKG2D T cells also protected against the growth of heterogeneous tumours comprising both ligand‐positive and ‐negative cells, even upon rechallenge of tumour‐free mice [54]. In a model of glioblastoma, chNKG2D T cells trafficked to the tumour site when injected intravenously (i.v.), although intratumoral injection significantly enhanced their infiltration [55]. Reduction of tumour burden was followed by long‐term disease protection, with prior cranial irradiation synergistically enhancing this effect. These T cells were shown to survive longer within the brain than other CAR T cells [55]. chNKG2D CAR T cells also proved potently cytotoxic against pancreatic, colorectal and leukaemic cell lines *in vitro*, secreting high levels of IFN‐γ. In a murine xenograft model established using the pancreatic PANC‐1 cell line, a dramatic reduction in tumour burden was observed after treatment [17].

The targeting capacity of NKG2D was further exploited in the design of second‐ and third‐generation CARs. Both CD4^+^ and CD8^+^ T cells expressing NKG2D fused to CD28 and CD3ζ (Fig. 2b) lysed MICA‐positive Ewing’s sarcoma tumour cells, accompanied by secretion of TNF‐α and FasL expression [56]. Notably, T cells transduced with a similar construct incorporating the 4‐1BB co‐stimulatory component were shown to self‐enrich (Fig. 2b) [57]. These T cells also efficiently eliminated ovarian cancer cell lines in which NKG2DL expression had been further enhanced using the histone deacetylase inhibitor, sodium valproate [57]. Spontaneous enrichment in culture was also reported for three NKG2D‐targeted CARs in which signalling was provided by CD3ζ alone (NKG2Dz) or together with a 4‐1BB (NKG2D‐BBz) or CD27 (NKG2D‐27z) co‐stimulatory module (Fig. 2b) [58]. As these T cells expressed low levels of T cell immunoglobulin and mucin domain‐containing protein‐3 (Tim‐3) and programmed cell death 1 (PD‐1) and lacked 4‐1BB, it was concluded that this phenomenon was due to fratricide or self‐stimulation rather than tonic signalling, which leads to exhaustion [58]. This fratricide can be attributed to activation‐dependent NKG2DL on T cells, which would trigger NKG2D CAR activation [59, 60]. Upon co‐culture with the triple‐negative breast cancer (TNBC) cell line, MDA‐MB‐431, these CAR T cells demonstrated cytotoxic activity, expression of activation markers and secretion of IFN‐γ, further enhanced by co‐stimulatory signalling [58]. In an MDA‐MB‐431 xenograft model, marginal efficacy was achieved with NKG2Dz, whereas NKG2D‐BBz or NKG2D‐27z CAR T cells caused more substantial disease regression [58].

T cells expressing NKG2D fused to 4‐1BB and CD3ζ (Fig. 2b) were further shown to kill glioblastoma stem cells (GSCs) within cell spheres, accompanied by secretion of proinflammatory cytokines [61]. When evaluated in glioblastoma xenograft‐bearing mice, tumour regression was accompanied by depletion of Nestin‐positive GSCs [61]. A similar CAR (Fig. 2b) was shown to efficiently destroy NKG2DL‐expressing gastric cancer cell lines, accompanied by cytokine release and lysosomal granule exocytosis [37]. When assessed in a derived xenograft model, CAR T cells accumulated within regressing tumours. Exposure to low‐dose cisplatin up‐regulated tumour NKG2DL expression, thereby increasing susceptibility to CAR T cell‐mediated destruction, accompanied by increased cytokine secretion [37]. Having demonstrated that NK cells target osteosarcoma tumour‐initiating cells through NKG2D, Fernández *et al*. engineered NKG2D‐4‐1BB‐CD3ζ CD45RA^−^ memory CAR T cells to treat this cancer [62]. These T cells demonstrated reduced alloreactivity when compared to CD45RA^+^ naive T‐cells, accompanied by increased cytolytic capacity. In an orthotopic osteosarcoma xenograft model, they significantly reduced disease burden and prolonged survival upon tumour rechallenge, without significant toxicity [62]. The same group also developed a good manufacturing practice (GMP)‐compliant large‐scale production protocol to generate NKG2D CAR T cell products for clinical trials, using the closed and automated CliniMACS Prodigy system [63].

## Preclinical assessment of safety of NKG2D‐targeted CARs

Mouse studies have provided some insights into the potential toxicity of NKG2D‐targeted CAR T cells in man. Activation of CAR T cells can result in life‐threatening cytokine release syndrome (CRS) and/or on‐target off‐tumour toxicity, whereby CAR T cells are activated by healthy cells [64]. In the case of NKG2D‐based CARs, risks are theoretically limited by the preferential expression of NKG2DLs on malignant and otherwise distressed cells. Moreover, the majority of preclinical studies that have evaluated NKG2DL‐targeted CARs (predominantly using C57BL/6‐based models) have demonstrated minimal toxicity. Importantly, however, it should be noted that there are substantial differences in NKG2DL expression between humans and mice, as summarized above. Moreover, NKG2DL expression can differ between individual mouse strains [35, 65]. Indeed, when NKG2D‐targeted CAR T cells were first evaluated in BALB/c mice, severe on‐target off‐tumour toxicity was unexpectedly uncovered [66]. T cells from BALB/c and C57BL/6 hosts were engineered to express three constructs; NKG2D fused to CD3ζ (NKz), the same construct with additional Dap10 (NK10z) and NKG2D fused to CD28 followed by CD3ζ (NK28z) (Fig. 2b). Cell surface CAR expression levels, cytolytic capacity and cytokine release were all greater when evaluated using BALB/c rather than C57BL/6 T cells, with highest levels seen using NK10z^+^ T cells [66]. In a BALB/c syngeneic mammary tumour model, marked toxicity was observed within hours of T cell infusion. Toxicity was most apparent using NK10z, milder with NK28z, while no significant toxicity was seen in NKz‐treated mice [66]. Similar observations were noted in naive tumour‐free mice. In contrast, less severe effects were observed in C57BL/6 mice, most prominently again with NK10z. Nonetheless, NK10z treatment resulted in a 25% lethality rate in C57BL/6 mice in that study [66]. Pretreatment with cyclophosphamide exacerbated these effects. Once again, toxicity was more apparent in BALB/c mice and was ascribed to severe CRS. Similar strain‐dependent differences have been observed with CD19‐targeted CAR T cells [67, 68]. Furthermore, pulmonary immunopathology occurred and was attributed to enhanced NKG2DL transcription following cyclophosphamide conditioning [66].

A subsequent dose‐escalation study using C57BL/6 mice reported only limited treatment‐associated toxicity (primarily CRS) at the highest dose of 2 × 10^7^ chNKG2D CAR T cells (Fig. 2a) [69]. Although substantial T cell numbers were present in the lungs, this was attributed to a first‐pass effect following i.v. delivery. No significant histological damage was observed in lungs or other major organs. Moreover, given the requirement for IFN‐γ for anti‐tumour activity but not toxicity, this model demonstrates that therapeutic efficacy and CRS are not necessarily interlinked [69]. Importantly, transient toxicity attributable to initial administration of CAR T cells was not seen with subsequent doses. Consequently, it was concluded that repeat administration of lower CAR T cell doses could limit toxicity while maintaining efficacy [69]. Similar findings were reported in models of multiple myeloma and ovarian cancer [39, 53].

## Clinical evaluation of NKG2D‐targeted CAR T cells

The preclinical data summarized above (see also [70]) paved the way for clinical assessment of NKG2D‐targeted CAR T cell immunotherapy by the biotechnology company, Celyad. The first‐in‐man Phase I clinical trial employed the original chNKG2D CAR [11], later renamed CM‐CS1, NKR‐2 and then CYAD‐01. This study had a 3 + 3 design and involved the treatment of patients at four dose levels (1 × 10^6^, 3 × 10^6^, 1 × 10^7^ and 3 × 10^7^ autologous CAR T cells), administered i.v.V as a fresh product without lymphodepletion [71, 72]. Patient diagnoses were relapsed/refractory acute myeloid leukaemia (r/r AML), myelodysplastic syndrome (MDS) or multiple myeloma. Investigators concluded that therapy was well tolerated without dose‐limiting toxicities (DLTs). No protocol‐defined objective responses were noted, although many patients had stable disease (SD) over the ensuing 12 months on alternative treatments.

This study was followed by further dose‐escalation and expansion trials, which are currently still ongoing. The first, entitled THINK (Therapeutic Immunotherapy with NKR‐2), entailed the i.v. delivery of three infusions of autologous CYAD‐01 T cells at 2‐week intervals, without either lymphodepleting or bridging chemotherapy. Three dose‐levels were used; namely 1× 10^8^, 1 × 10^9^ and 3 × 10^9^ CAR T cells [73]. Seven clinical indications were selected; namely, AML, multiple myeloma and five solid tumours (pancreatic, urothelial, ovarian, colorectal and TNBC tumours). Within the haematological arm, CRS occurred in 13 of 25 patients and reached grades 3 (severe) and 4 (life‐threatening) in one case each, following treatment at dose‐levels 2 and 3, respectively. Both patients recovered following treatment with tocilizumab [74, 75]. Anti‐leukaemic effects were observed in eight patients, with three complete responses (CR), two partial responses (PR) and three patients with SD. The study concluded that CYAD‐01 exhibited a good overall safety profile with evidence of anti‐tumour activity [75]. More recently, the THINK protocol has been amended to evaluate a more frequent CAR T cell dosing schedule [76].

Subsequent clinical studies also incorporated chemotherapy to favour expansion of CAR T cells and benefit from additional anti‐tumour activity. In the SHRINK (Standard cHemotherapy Regimen and Immunotherapy with NKR‐2) study, colorectal cancer patients with potentially resectable liver metastases (neoadjuvant cohort) or non‐resectable metastatic disease (refractory cohort) received three i.v. doses of autologous CYAD‐01 at 2‐week intervals. CAR T cells were administered on day 3 of concurrently administered FOLFOX chemotherapy (FOlinic acid, 5‐Fluorouracil, OXaliplatin) [77]. Dose escalation proceeded through 1, 3 and 10 × 10^8^ CAR T cells per infusion. No DLTs were observed in nine patients. One patient in the neoadjuvant cohort achieved a PR, while two achieved SD, and in the refractory cohort, four of five patients achieved SD [77].

The DEPLETHINK (LymphoDEPLEtion and Therapeutic Immunotherapy with NKR‐2) clinical trial evaluated the dose‐escalation of autologous CYAD‐01 in patients presenting with r/r AML or MDS. Dose escalation proceeded through 1, 3 and 10 × 10^8^ CAR T cells, administered as a single infusion after CyFlu (cyclophosphamide 300 mg/m^2^ and fludarabine 30 mg/m^2^, each for 3 days) preconditioning. The original CYAD‐01 product manufacturing process, dubbed ‘mAb process’, included a blocking NKG2D antibody to inhibit T cell fratricide, yielding large numbers of differentiated T cells with potent cytolytic activity but limited persistence [78]. To enrich for less differentiated cells, Celyad developed the ‘OptimAb’ process, which includes an 8th‐day culture period, with the addition of an NKG2D blocking antibody and a selective PI3K inhibitor [78]. OptimAb manufactured T cells produced higher cytokine levels upon activation and demonstrated improved anti‐tumour activity in a preclinical AML model. Initially, nine patients were enrolled over the first two dose‐levels, receiving monoclonal antibody (mAb) processed T cells. Thereafter, further patients were recruited at the second dose‐level using OptimAb‐processed T cells. No objective responses were observed in the first nine treated patients, although three of these patients did not exhibit disease progression over 1 month [79]. Moreover, the safety profile was generally favourable, with cases of grades 3 and 4 CRS reported, both of which responded to Tocilizumab. Dose‐dependent T cell engraftment was noted following CyFlu preconditioning [80].

The SHRINK study provided the basis to design alloSHRINK (Standard cHemotherapy Regimen and Immunotherapy with allogeneic NKG2D‐based CYAD‐101 chimeric antigen receptor T cells). AlloSHRINK entails the administration of three consecutive doses of allogeneic (healthy donor‐derived) CYAD‐01 with concurrent FOLFOX chemotherapy. This was the first trial to assess allogeneic non‐gene‐edited CAR T cell immunotherapy in a solid tumour setting [81, 82]. T cells were engineered to co‐express a TCR inhibitory molecule (TIM), an endodomain truncated version of CD3ζ, designed to minimize risk of graft‐*versus*‐host disease (GvHD). Dose escalation proceeded through 1, 3 and 10 × 10^8^ CAR T cells per infusion. Six of 12 patients enrolled in the dose‐escalation phase experienced at least one adverse reaction. However, toxicities were only grades 1 and 2, and no DLTs or GvHD was reported. Two patients achieved a PR (one durable for > 6 months) and seven maintained SD. Moreover, CYAD‐01 cells were still detectable 40 days after the infusion [81, 82].

Recently, Celyad launched another trial, designated CYCLE‐1, in which they are evaluating a new OptimAb‐processed product termed CYAD‐02. This CAR further incorporates an shRNA targeting MICA and MICB, aimed to increase *in‐vitro* expansion and engraftment of CAR T cells. Patients with r/r AML or MDS will be enrolled and will receive CyFlu preconditioning [83].

## The arsenal of natural cytotoxicity receptors (NCR) and their use in CAR design

The NCR family consists of three type I transmembrane receptors; namely, NKp30, NKp44 and NKp46, encoded by the *NCR3, NCR2* and *NCR1* genes, respectively [21, 84, 85, 86]. While NKp30 and NKp46 are constitutively expressed on NK cells, NKp44 is only expressed upon activation [23, 87]. Although structurally distinct, the NCRs consist of one (NKp44 and NKp30) or two (NKp46) immunoglobulin (Ig)‐like extracellular domains and a charged transmembrane domain, enabling these receptors to associate with adaptor molecules that initiate downstream signalling [21, 88]. All three NCRs recognize ligands found on cancerous and virally infected cells, thereby promoting NK cell activation and cytolytic granule release. Depending on the isoform expressed and engaged ligand, some NCRs may also exert inhibitory effects [89]. Murine NK cells only express *NCR1* (NKp46), whereas *NCR3* (NKp30) is a pseudogene and *NCR2* (NKp44) is not expressed [90]. Information about NCR ligands remains limited in comparison to NKG2DLs. Nonetheless, several have been identified, prompting the evaluation of NCR binding units in the targeting of CAR T cell specificity [88, 89].

## NKp30 and derived CARs

**Fig. 3:**
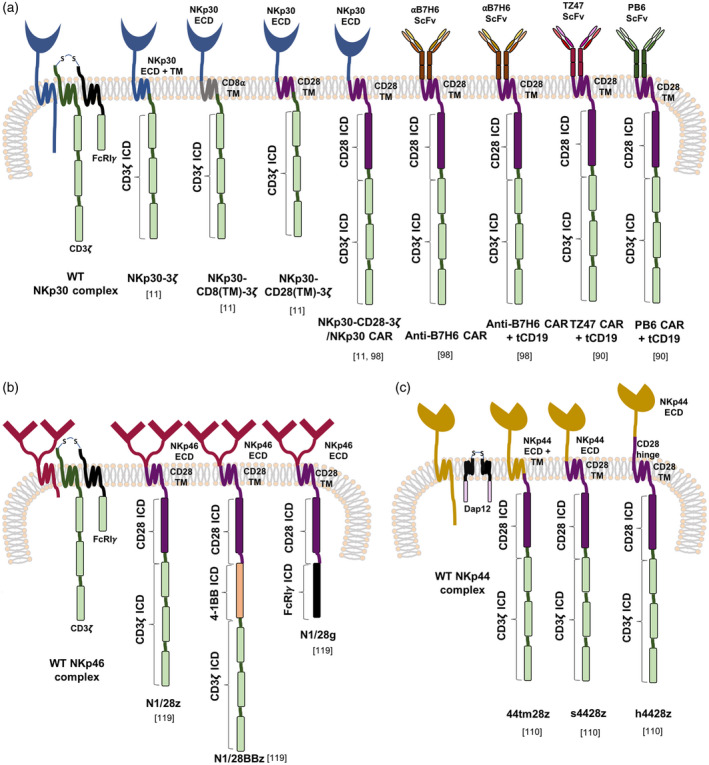
Chimeric antigen receptors (CAR) employing the targeting domains of NCRs. Natural cytotoxicity receptors (NCRs) target a wide range of tumour‐associated and viral ligands, and primarily mediate activating signalling in natural killer (NK) cells through the adaptor proteins, FcRI*γ*, CD3ζ and DNAX‐activating protein (Dap)12. These targeting components have been utilized in both first‐ and second‐generation CARs that target NKp30 ligands (a), NKp46 ligands (b) and NKp44 ligands (c). WT = wild‐type; ICD = intracellular domain; ECD = extracellular domain; TM = transmembrane.

NKp30 is a member of the CD28 receptor family [90]. The *NCR3* gene is located within the MHC class III region and can give rise to six alternatively spliced transcripts, namely NKp30a–NKp30f. NKp30a and NKp30b are activating receptors, while NKp30c plays an immunosuppressive role. All three isoforms have a V‐type immunoglobulin (Ig) domain. NKp30d–NKp30f are expressed less frequently and encode a C‐type extracellular Ig domain [29, 91, 92]. A charged arginine residue within the NKp30 transmembrane domain allows association with CD3ζ or FcRI*γ* adaptor units to initiate activating signalling (Fig. 3a) [14, 84]. Two NKp30 ligands have been identified; namely, the cell surface molecule, B7H6, and the nuclear protein, BAT3 (also known as BAG6) (Fig. 1a). Cell surface expression of both ligands is up‐regulated upon DNA damage and cellular/endoplasmic reticulum stress, with BAT3 also expressed on certain immune cells [20, 29, 88, 93]. B7H6 plays a role in inflammation and tumour surveillance. Enhanced B7H6 expression is found in a range of tumours [93, 94], with further up‐regulation by radiation, chemotherapy and immunotherapy [95]. NKp30 recognizes B7H6 through the complementarity determining region (CDR)‐like loops of its Ig variable domain with an affinity of 1000 nM (K_D_), whereby homo‐oligomerization is believed to enhance avidity [90, 93, 96, 97]. Soluble forms of both B7H6 and BAT3 may be released through the action of metalloproteases, and these can inhibit NK cell activity, accompanied by poorer overall survival [93, 98, 99, 100, 101]. Moreover, an additional soluble inhibitory ligand, Galectin‐3, was shown to promote tumour escape by inhibiting NK cell‐mediated tumour cell lysis [102].

Given this background, NKp30 has been incorporated into a number of tumour‐specific CARs. As T cells lack FcRI*γ* required for downstream NKp30 signalling, chimeric receptors were engineered in which the extracellular domain of NKp30 was fused to CD3ζ alone or in combination with CD28, providing first‐ and second‐generation CAR designs (Fig. 3a) [14]. Anti‐tumour activity was shown to be ligand‐ and PI3K‐dependent [14]. T cells that expressed the CD28‐containing CAR also exhibited increased proliferation, IL‐2 release and expression of the anti‐apoptotic protein, Bxl‐X_L_ [14]. In a murine systemic lymphoma model, NKp30–CD28–CD3ζ CAR T cells induced 17% long‐term survival accompanied by epitope spreading, indicated by rejection of rechallenge with ligand‐negative tumour [14].

An obstacle to the use of NKp30‐targeting CAR T cells is the expression of cell surface BAT3 on immature DCs (iDCs) and some monocytes [93], rendering these cells sensitive to NKp30 CAR T cells [14]. To circumvent this, an scFv‐based B7H6‐specific CAR (CD28 + CD3ζ endodomain) was engineered (Fig. 3a) [103]. B7H6‐specific CAR T cells demonstrated anti‐tumour activity *in vitro* and in models of systemic lymphoma and ovarian cancer [103]. Fully human CARs targeted against B7H6 have also been described [95].

## NKp46 and derived CARs

NKp46 is a 46 kDa type I membrane spanning receptor and, like NKp30, it associates with CD3ζ and FcRI*γ* chains [21]. Highlighting its conserved role in immune surveillance, this is the only NCR gene with an orthologue in mice and other species [104]. It is comprised of a short endodomain, a hydrophobic stalk and an ectodomain composed of two C2‐type Ig domains connected via a hinge, contrasting with the single V‐type Ig segment of NKp30 (Fig. 3b) [105]. Similarly to NKp30, it is expressed on all NK cells regardless of activation state, and directs the killing of transformed cells [106, 107]. The ligand(s) recognized by NKp46 remains ambiguous, with a few proposed cellular and pathogen‐associated candidates. Along with both NKp30 and NKp44, NKp46 has been reported to bind cell surface‐associated heparan sulphate proteoglycans (HSPG), albeit as accessory co‐factors rather than directly activating ligands [89, 108, 109]. Nonetheless, this may also contribute to a ‘tumour‐sensing’ activity, given that post‐translational modification of HSPG is commonly altered in transformed cells.

Several viral haemagglutinins have also been proposed as ligands of NKp46 and NKp44. Binding is dependent on the sialylation state of these NCRs [110, 111]. It was further shown that *Mycobacterium tuberculosis*‐infected monocytes secrete another putative NKp46 ligand, vimentin. Cancer cells have also been known to up‐regulate cell surface expression of vimentin, potentially playing a role in epithelial–mesenchymal transition (EMT) [107, 112]. Other tumour‐specific cell surface targets for NKp46 remain to be elucidated. Nevertheless, it is evident that NKp46 plays a crucial role in the elimination of tumour and virally infected cells, probably due to the recognition of stress ligands [104, 113, 114].

To date, one study has assessed anti‐tumour activity of second‐ and third‐generation NKp46‐targeted CAR T cells [115]. The extracellular component of NKp46 was fused to a CD28 spacer and transmembrane segment followed by the signalling domains of CD28 plus CD3ζ (N1/28z), CD28 plus FcRI*γ* (N1/28g) or CD28 plus 4‐1BB, followed by CD3ζ (N1/28BBz) (Fig. 3b). N1/28z was selected as the best‐performing CAR, based on *in‐vitro* studies using Jurkat and primary human T cells. Anti‐tumour activity was also demonstrated using a chick embryo chorioallantoic membrane tumour model and against metastatic cervical cancer (Henrietta Lacks; HeLa) tumour xenografts [115].

## NKp44 and derived CARs

The *NCR2* gene encoding NKp44 is located within the class III region of the MHC along with *NCR3* (NKp30), suggesting an inter‐relatedness between these receptors [104]. This 44 kDa type I transmembrane protein contains a single V‐like Ig domain and a transmembrane segment which mediates an interaction with DNAX‐activation protein 12 (Dap12) for downstream activating signalling [29]. *NCR2* can be expressed as three splice variants. Transcripts‐2 and ‐3 bear a short intracellular tail while the longer endodomain of transcript‐1 contains an ITIM, potentially enabling this isoform to relay both activating and inhibitory signals [29, 116, 117, 118]. The tumour cytokine milieu and hypoxic microenvironment has also been reported to influence cell surface expression and the activating or inhibitory outcome of NKp44 signalling [116]. An NKp44 ligand was first identified on human immunodeficiency virus‐1‐infected CD4^+^ T cells, subsequently characterized as an isoform of mixed‐lineage leukaemia protein 5 (MLL5), a predominantly nuclear protein that regulates the cell cycle [119]. However, this isoform bears a unique C‐terminal sequence, allowing it to shuttle via the endosomal pathway to the tumour cell surface [24, 116]. NKp44 was also shown to induce NK cell activation through the recognition of platelet‐derived growth factor DD, inhibiting cancer cell proliferation and releasing proinflammatory cytokines [120]. Several inhibitory ligands have also been identified for NKp44. Exemplifying this, proliferating cell nuclear antigen (PCNA) is a nuclear protein that regulates DNA replication. Within malignant cells, it can shuttle into exosomes or to the plasma membrane via a PCNA/human leucocyte antigen‐1 (HLA‐1) complex, which interacts with the inhibitory NKp44‐1 isoform [121, 122]. Furthermore, the extracellular matrix protein, nidogen‐1, is released by the proteolytic cleavage of cathepsin‐S on tumour cells, inhibiting NK cells and facilitating immune evasion [123]. Several viral and bacterial ligands have also been identified, as reviewed in [29].

Similarly to NKp46, a single study has been undertaken which evaluated three NKp44‐based CARs. These comprised a fusion of the NKp44 extracellular domain to the transmembrane and endodomain of CD28 followed by that of CD3ζ (s4428z), an identical construct further incorporating a CD28 hinge/spacer to increase the distance between the targeting domain and the plasma membrane (h4428z), and finally a CAR, whereby the extracellular and transmembrane domain of NKp44 were joined to a fused CD28–CD3ζ endodomain (44tm28z) [124] (Fig. 3c). The s4428z CAR was subsequently chosen as the lead construct based on superior IFN‐*γ* secretion. These T cells also demonstrated high levels of cytotoxicity, secretion of IL‐2 and TNF‐α and expression of 4‐1BB upon activation with NKp44 ligand‐positive cell lines. Anti‐tumour activity was also confirmed in primary human melanoma co‐cultures and in HeLa and MDA‐MB‐435 NSG mouse xenograft models [124].

## Can alternative NK receptors be used to generate CARs?

In addition to NKG2D and the NCRs, NK cells express a host of alternative innate receptors which can induce cellular activation and play a role in anti‐tumour immunity. Notable examples are DNAM‐1 and 2B4. The NK cell surface glycoprotein DNAM‐1 (CD226) is a 65kDa activating receptor of the Ig‐superfamily, is constitutively expressed by most NK cells, αβ and γδ T cells, monocytes and by a subset of B cells [125, 126]. It is highly expressed on CD8^+^ T cells and is up‐regulated on CD4^+^ T cells upon activation [127, 128]. Primarily implicated as an adhesion molecule, which physically associates with the integrin lymphocyte function‐associated antigen 1 (LFA‐1), it plays a crucial role in immune synapse formation and in NK and CD8^+^ T cell cytotoxicity. DNAM‐1 recognizes two immunoglobulin adhesion receptors, namely CD155 (PVR; poliovirus receptor) and CD112 (Nectin‐2; PVR‐related protein 2), with an affinity of 230 nM and 310 nM, respectively [129]. While both PVRs are expressed at low levels in various normal tissues, they are up‐regulated on tumour cells [130, 131, 132]. However, both PVRs are also bound by the inhibitory NK receptor, T cell immunoglobulin and ITIM domain (TIGIT). In addition, CD96 (T cell activation increased late expression; TACTILE) binds CD155, leading to disputed consequences. Moreover PVR‐related Ig domain, (PVRIG) binds CD112, leading to inhibitory signalling. These DNAM‐1‐competing receptors (TIGIT in particular) have higher affinity for PVRs and consequently have also been proposed as targets for immune checkpoint inhibition [133, 134, 135]. DNAM‐1 has further been shown to synergize with other activating NK cell receptors, promoting cellular activation through an ITT‐like motif, and inducing effector cytokine production [136, 137, 138]. Although an activating receptor in NK cells, DNAM‐1 mainly acts as a co‐stimulator in T cells [126, 127, 139]. Consequently, the engineering of a stimulatory CAR that incorporates the DNAM‐1 or TIGIT targeting domain could serve as an alternate strategy to target tumour cells. In support of this, DNAM‐1 was implicated in the lysis of tumours that lack expression of ligands for other activating NK receptors [140].

**Fig. 4:**
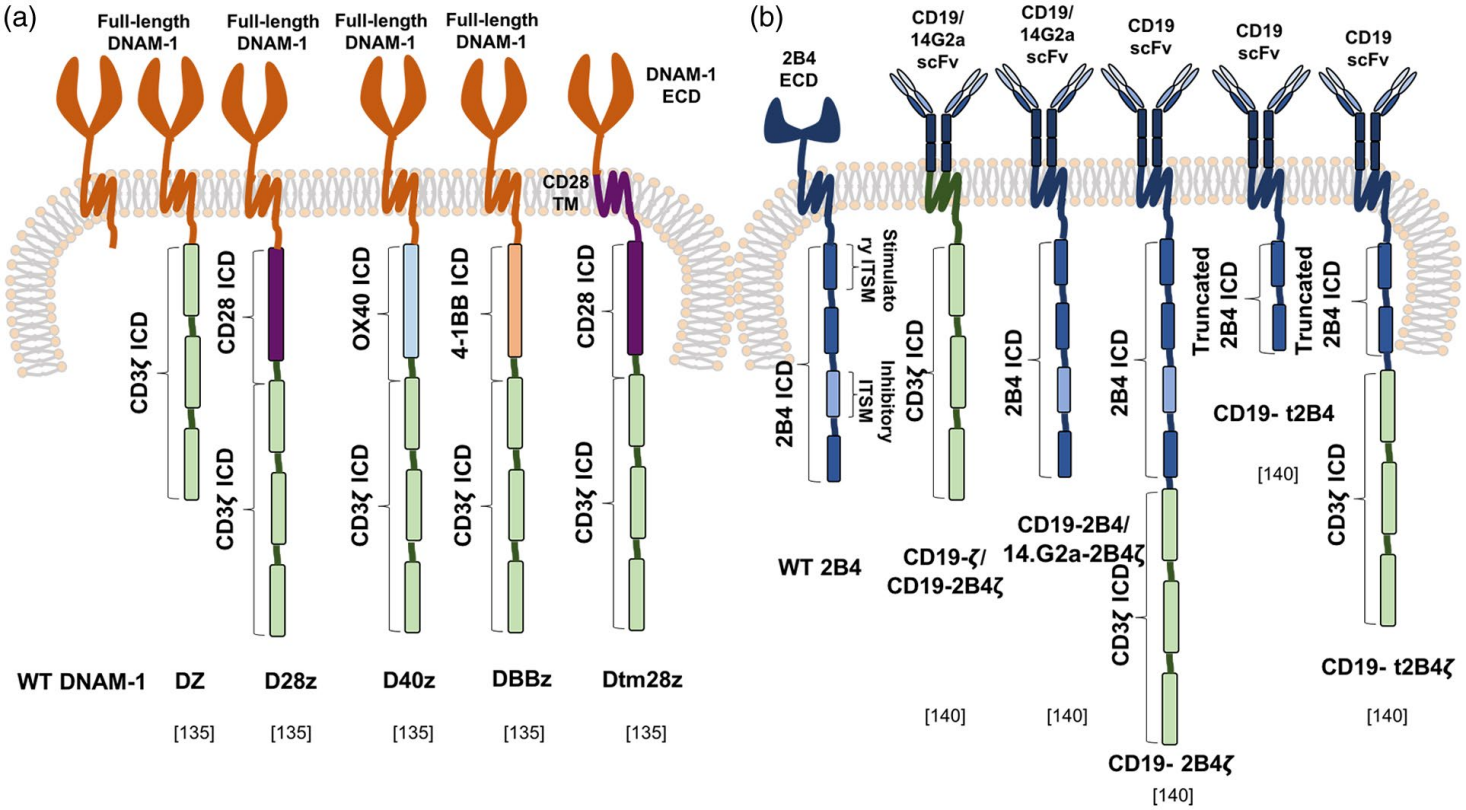
Chimeric antigen receptor (CAR)‐mediated therapy using alternative endogenous natural killer (NK) cell receptors. In addition to natural killer group 2D (NKG2D) and the natural cytotoxicity receptors (NCRs), several alternative NK cell receptors and co‐receptors have been implicated in anti‐tumour immunity. CARs based on the DNAX accessory molecule‐1 (DNAM‐1) targeting domain (a) and 2B4 signalling components (b) have been assessed for their ability to lyse target tumour cells.

One study has assessed DNAM‐1‐based CAR T cells [141]. Constructs comprised of unmodified DNAM‐1, full‐length DNAM‐1 fused to CD3ζ (DZ) or CARs that additionally included a CD28 (D28Z), OX40 (D40Z) or 4‐1BB (DBBZ) endodomain, placed downstream of DNAM‐1. The final construct incorporated only the extracellular component of DNAM‐1, fused to the transmembrane and cytoplasmic portion of CD28, followed by CD3ζ (Dtm28Z) (Fig. 4a) [141]. Cell surface expression of DZ was highest, which correlated with superior tumour cytolytic ability. However, only low levels of IFN‐γ were produced upon co‐culture with tumour cells lines. When DZ T cells were administered intratumorally in a syngeneic melanoma murine model, tumour burden was reduced [141].

One concern with DNAM‐1‐based CARs is the fact that PVRs are expressed on some normal cell types. Indeed, several of the DNAM CARs described above induced perforin‐ and GM‐CSF‐dependent CRS when infused into C57BL/6 mice [69]. One potential solution entails the use of a PVR‐targeting ligand to direct the specificity of a co‐stimulatory switch receptor (CSR). Illustrating this, a TIGIT‐based CSR was expressed in human T cells and delivered co‐stimulation through a coupled CD28 endodomain, even in the presence of the immunosuppressive cytokine, TGF‐β [135]. T cells expressing TIGIT‐28 also significantly reduced tumour burden and lengthened survival in a melanoma xenograft model [135].

Another noteworthy NK receptor is the signalling lymphocyte activation molecule (SLAM) family member, 2B4, encoded by *CD244*. This multi‐functional NK immunomodulatory receptor is expressed on many haematopoietic cells, including NK cells, γδ T cells, basophils, monocytes and a proportion of αβ CD8^+^ T cells, MDSCs and DCs. It has an extracellular domain that contains two Ig loops which bind CD48, both in *cis* and in *trans*, at an affinity of 8000 nM (K_D_) [142]. CD48 is ubiquitously expressed in haematopoietic cells and there is some evidence implicating this receptor in NK cell‐mediated cytotoxicity of tumour cells [143, 144]. The 2B4 cytoplasmic domain contains four immunoreceptor tyrosine‐based switch motifs (ITSMs) which can interact with activating (e.g. SLAM‐associated protein; SAP) or inhibitory (e.g. phosphatase‐containing) signalling partners, the balance between which dictates outcome [137, 145]. 2B4 has been shown to synergize with DNAM‐1, NKp46 and NKG2D [27]. Its endodomain has been evaluated in the context of CAR NK cells. One study assessed the activity of CD19‐ or GD2 ganglioside‐targeted CARs which incorporated the full 2B4 cytoplasmic domain with the four ITSMs, either alone (2B4) or fused to CD3ζ (2B4ζ) [146]. In addition, constructs were engineered with a truncated 2B4 cytoplasmic domain that incorporated the first two activating ITSMs alone (t2B4 and t2B4ζ) (Fig. 4b). The CAR NK cells incorporating both (truncated or intact) 2B4 and CD3ζ demonstrated significantly enhanced activation and secretion of IFN‐γ and TNF‐α upon stimulation with target cells, in comparison to those with only one component. 2B4 signalling alone induced limited NK activation and secretion of cytokines; however, it prompted sufficient degranulation to elicit leukaemic cell killing [146]. More recently, a mesothelin‐specific CAR has been engineered to contain the NKG2D transmembrane domain coupled to a fusion of the 2B4 and CD3ζ endodomain. Following expression in induced pluripotent stem cells, transduced cells were differentiated into NK cells. Impressive anti‐tumour activity was demonstrated *in vitro* and *in vivo* [147]. Additionally, 2B4 co‐stimulation resulted in superior anti‐tumour efficacy of CD5‐targeted CAR NK cells against T cell leukaemia compared to 4‐1BB co‐stimulated cells. This strategy was used to circumvent fratricide observed using CD5‐targeted CAR T cells [148].

## Conclusions

The activating NK cell receptors have been evolutionarily designed to mediate optimal signalling, fine‐tuned through balancing of distinct activating and inhibitory pathways. Moreover, they have evolved to recognize a variety of specific surface antigens, ensuring the destruction of infected or malignant cells, but sparing healthy tissue. These characteristics make activating NK receptors attractive candidates for application in tumour immunotherapy. Chimeric antigen receptor‐engineered T cells provide a favourable platform to evaluate this concept, coupling the antigen recognition capabilities of these NK receptors to the delivery a ‘bespoke’ T cell activating signal, resulting in effector cytokine production, recruitment of innate immune cells, tumour cell lysis and, importantly, the generation of a memory response. The latter may ultimately prove crucial to success, particularly in the arena of solid tumours, given that this may hinder the emergence of any *de‐novo* tumour variants. The concern for potential on‐target off‐tumour toxicity owing to ligand expression in healthy organs warrants further evaluation to demonstrate the safety of this approach, particularly under circumstances in which coincident pathology occurs in vital organs. Nevertheless, experience gathered to date across a range of preclinical and clinical studies using NKG2D targeted CARs provides strong encouragement for the further development of these therapies.

## Disclosure

J. M. is chief scientific officer, shareholder and scientific founder of Leucid Bio, which is a spinout company focused on development of cellular therapeutic agents. D. M. D. is a consultant to Leucid Bio. There are no additional competing financial interests or conflicts of interest to declare.
